# Glycaemic Control, Blood Pressure and Lifestyle Behaviours in Relation to Sarcopenia Among Older Adults in Korea: A Population‐Based Cross‐Sectional Study

**DOI:** 10.1111/ajag.70174

**Published:** 2026-04-29

**Authors:** Kyeongmin Jang

**Affiliations:** ^1^ Department of Nursing Daejin University Pocheon South Korea

**Keywords:** aged, blood pressure, glycosylated haemoglobin, motor activity, sarcopenia

## Abstract

**Objective:**

The aim of this study was to estimate the prevalence of sarcopenia and examine its associations with glycaemic control, blood pressure and lifestyle behaviours among older adults in Korea.

**Methods:**

We conducted a secondary analysis of the 2023 Korea National Health and Nutrition Examination Survey that included 1394 adults aged 65 years or older. Sarcopenia followed Asian Working Group for Sarcopenia 2019 criteria (low appendicular skeletal muscle mass index and low handgrip strength). Complex‐sample descriptive tests and multivariable logistic regression identified independent correlates adjusting for sociodemographic, behavioural and clinical covariates. Blood pressure was measured per standard protocol; fasting glucose and haemoglobin A1c (HbA1c) were assayed in a central laboratory.

**Results:**

Sarcopenia prevalence was 14% (*n* = 199). Age 76 years or older (OR 3.19, 95% CI 2.27–4.49) and poor glycaemic control (HbA1c ≥ 7.0%; OR 1.89, 95% CI 1.16–3.08) were associated with higher odds of sarcopenia. Protective factors included body mass index ≥ 25 kg/m^2^ (OR 0.32, 95% CI 0.21–0.48), meeting the World Health Organization physical activity guideline (OR 0.50, 95% CI 0.28–0.90) and alcohol consumption in the past year (OR 0.58, 95% CI 0.41–0.82). Hypertension diagnosis and smoking were not significant. Diastolic, but not systolic, blood pressure was lower among participants with sarcopenia.

**Conclusions:**

In a nationally representative Korean sample, sarcopenia was common and linked to modifiable factors—glycaemic control and physical activity. Findings support integrating HbA1c monitoring, resistance and aerobic exercise promotion, and nutrition counselling into community and primary care to reduce sarcopenia risk and support healthy ageing.

## Abstract (highlights)

**Practice Impact:**

Routine grip strength testing and simple body composition checks, combined with HbA1c monitoring and tailored exercise/nutrition counselling, can be embedded into community and primary care workflows to identify at‐risk older adults early and mitigate sarcopenia‐related functional decline.

**Policy Impact:**

Population strategies that incentivise diabetes control and physical activity in later life—through primary care quality indicators, community exercise programs and nutrition support—may lower sarcopenia burden and preserve functional independence in ageing societies.

## 1 Introduction

Sarcopenia, a progressive and generalised loss of skeletal muscle mass and strength, is a major contributor to physical disability, poor quality of life and mortality among older adults [1, 2]. In Korea, sarcopenia has also been associated with adverse outcomes, such as falls, mobility limitation and mortality, underscoring its clinical and public health importance [3]. With global ageing accelerating, sarcopenia has become an increasingly important public health concern. In South Korea, the proportion of adults aged 65 years or older is projected to exceed 20% in 2025, marking the transition to a super‐aged society, and to exceed 30% by 2036 [4]. These demographic changes are expected to substantially increase the burden of age‐related conditions, such as sarcopenia and functional decline. In response, the Asian Working Group for Sarcopenia (AWGS) updated its diagnostic criteria in 2019, emphasising that both low muscle mass and low strength are core components [5].

The pathophysiology of sarcopenia is multifactorial, involving hormonal changes, chronic inflammation, malnutrition, physical inactivity and chronic diseases [6]. Among these, diabetes has emerged as a key contributor: Insulin resistance and hyperglycaemia can accelerate muscle catabolism, whereas reduced muscle mass exacerbates glucose dysregulation, creating a harmful cycle [6]. In individuals with diabetes, sarcopenia has been associated with poorer glycaemic control, reduced mobility and a higher risk of complications [7].

The role of hypertension in sarcopenia is less well established. Some evidence suggests that vascular dysfunction and impaired muscle perfusion associated with elevated blood pressure may contribute to muscle loss [8]; however, other studies report no significant relationship between sarcopenia and physician‐diagnosed hypertension [9]. Few investigations have examined associations with actual blood pressure values (systolic and diastolic) in older adults.

Lifestyle behaviours are also implicated in sarcopenia risk. Physical inactivity is a modifiable factor strongly associated with muscle decline. The World Health Organization (WHO) recommends that older adults engage in at least 150 min of moderate‐intensity aerobic physical activity per week to maintain musculoskeletal health [10]. Yet, many do not meet these recommendations, often due to age‐related limitations. Alcohol consumption and smoking have shown inconsistent associations with muscle mass, warranting further investigation.

Socioeconomic conditions may influence sarcopenia through differences in nutrition, health behaviours and access to care. Studies indicate that lower education and income are associated with poorer diet and physical inactivity—key risk factors for muscle loss in later life [11]. In addition, body mass index (BMI) remains a clinically relevant marker, and recent literature also highlights concerns regarding sarcopenic obesity [12, 13].

Despite increasing interest, few nationally representative studies in Korea have examined sarcopenia in relation to glycaemic control, blood pressure, health behaviours and socioeconomic factors. Existing evidence remains fragmented because of limited sample sizes, inconsistent diagnostic criteria and the use of non‐representative samples [14, 15]. Although recent KNHANES‐based studies have provided important population‐level data, further investigation of modifiable correlates is warranted [15]. Accordingly, this study used data from the 2023 Korea National Health and Nutrition Examination Survey (KNHANES) to (1) estimate the prevalence of sarcopenia among older adults in Korea; (2) examine its associations with glycaemic control indicators (fasting glucose and haemoglobin A1c [HbA1c]); (3) investigate differences in blood pressure and hypertension status by sarcopenia; and (4) identify sociodemographic, behavioural and clinical factors independently associated with sarcopenia.

## 2 Methods

### 2.1 Study Design and Data Source

This was a secondary analysis of nationally representative, cross‐sectional data from the 9th Korea National Health and Nutrition Examination Survey (KNHANES, 2022–2024). The KNHANES, administered annually by the Korea Disease Control and Prevention Agency, assesses the health and nutritional status of the non‐institutionalised Korean population using a complex, multistage, stratified cluster sampling design, with health interviews, physical examinations and nutrition surveys conducted by trained personnel under standardised protocols [16]. We used publicly available, anonymised raw data from the 2023 wave, released in December 2024.

### 2.2 Participants

Of 6929 participants in the 2023 KNHANES, 1836 were aged 65 years or older. We included 1394 older adults after excluding cases with missing data for key variables (skeletal muscle mass, handgrip strength, glycaemic markers, blood pressure, physical activity and covariates), yielding a complete‐case analytic sample.

### 2.3 Measurement of Sarcopenia

Sarcopenia was defined per the Asian Working Group for Sarcopenia 2019 guideline: concomitant low muscle mass and low muscle strength [5]. Appendicular skeletal muscle mass (ASM) was estimated via bioelectrical impedance analysis; the ASM index (kg/m^2^) was ASM divided by height squared. Low muscle mass was defined as ASM index < 7.0 kg/m^2^ for men and < 5.4 kg/m^2^ for women. Muscle strength was assessed by digital handgrip dynamometry under standardised procedures; low strength was defined as < 28.0 kg for men an*d* < 18.0 kg for women.

### 2.4 Independent Variables

Sociodemographic variables were age (continuous and dichotomised as < 76 vs. ≥ 76 years), sex, education (≤ elementary school vs. ≥ middle school) and household income (lowest quartile vs. other). Behavioural variables were current smoking (yes/no), alcohol consumption in the past year (yes/no) and meeting the WHO physical activity guidelines (≥ 150 min of moderate‐intensity aerobic activity per week) [10] Clinical/anthropometric factors were BMI (< 25 vs. ≥ 25 kg/m^2^), physician‐diagnosed hypertension and diabetes (yes/no), fasting glucose (mg/dL) and haemoglobin A1c (HbA1c; continuous and < 7.0% vs. ≥ 7.0%).

### 2.5 Blood Pressure and Glycaemic Control Measurements

Blood pressure was measured three times with a mercury sphygmomanometer under standardised conditions; the mean of the second and third readings defined systolic (SBP) and diastolic (DBP) blood pressure. Fasting venous samples (≥ 8 h) were analysed at a central laboratory for glucose and HbA1c using enzymatic methods.

### 2.6 Dichotomisation of Age

Receiver operating characteristic analysis (MedCalc, version 23.1.3; MedCalc Software Ltd., Ostend, Belgium) identified the optimal age threshold for sarcopenia discrimination. The area under the curve was 0.705 (95% CI 0.680–0.728, *p* < 0.001). The Youden index indicated an optimal cut‐off of older than 75 years; thus, age 76 years or older was used as a binary covariate (sensitivity 57.3%; specificity 74.4%).

### 2.7 Statistical Analysis

We described participant characteristics using descriptive statistics. Group differences by sarcopenia status were assessed with independent‐samples *t*‐tests (continuous variables) and χ^2^ tests (categorical variables). Multivariable logistic regression estimated adjusted odds ratios (ORs) and 95% confidence intervals (CIs) for factors associated with sarcopenia. Variable selection was guided by prior literature and conceptual relevance. Statistical significance was set at *p* < 0.05 (two‐sided). Analyses were performed using IBM SPSS Statistics for Windows, version 29.0 (IBM Corp., Armonk, NY, USA).

### 2.8 Ethical Considerations

This study used publicly available, de‐identified data from the 2023 Korea National Health and Nutrition Examination Survey (KNHANES). The KNHANES 2023 survey protocol was reviewed and approved by the Institutional Review Board of the Korea Disease Control and Prevention Agency (IRB No. 2022‐11‐16‐R‐A), and all participants provided written informed consent. Because this study was a secondary analysis of anonymised public data, additional institutional review board approval was not required.

## 3 Results

### 3.1 Participant Characteristics

The general characteristics of participants by sarcopenia status are shown in Table 1. Among the 1394 older adults included in the analysis, 199 (14%) had sarcopenia. Participants with sarcopenia were significantly older (75.35 ± 4.69 vs. 71.74 ± 4.85 years, *p* < 0.001) and had lower BMI, handgrip strength and ASM index than those without sarcopenia (all *p* < 0.001). Additionally, the sarcopenia group had a higher proportion of individuals with low educational attainment (*p* < 0.001), lower household income (*p* < 0.001), no alcohol consumption in the past year (*p* < 0.001) and not meeting the WHO physical activity guidelines (*p* < 0.001). There were no significant differences in sex, smoking status, physician‐diagnosed hypertension or diabetes, fasting glucose or HbA1c as a continuous variable.

**TABLE 1:** Comparison of characteristics between participants with and without sarcopenia (*n* = 1394).

Variable	No sarcopenia (*n* = 1195)	Sarcopenia (*n* = 199)	*t*/χ^2^	*p*
Demographic characteristics				
Age (years), mean ± SD	71.74 ± 4.85	75.35 ± 4.69	−10.02	< 0.001
Sex, male (%)	554 (46)	82 (41)	1.83	0.18
Socioeconomic factors				
Education level ≤ elementary (%)	482 (40)	107 (54)	12.62	< 0.001
Income (lowest quartile) (%)	495 (41)	117 (59)	20.90	< 0.001
Health behaviours				
Current smoker (%)	124 (10)	14 (7)	2.14	0.14
Alcohol consumption (past year) (%)	625 (52)	65 (33)	26.32	< 0.001
Meets WHO PA guideline (%)	198 (17)	14 (7)	12.03	< 0.001
Sarcopenia‐related measures				
BMI (kg/m^2^), mean ± SD	24.30 ± 3.03	22.25 ± 3.21	8.38	< 0.001
Hand grip strength (kg), mean ± SD	28.38 ± 7.94	18.77 ± 5.21	22.10	< 0.001
ASM index (kg/m^2^), mean ± SD	6.71 ± 0.92	5.77 ± 0.82	14.86	< 0.001

Abbreviations: ASM, appendicular skeletal muscle mass index; BMI, body mass index; PA, physical activity.

### 3.2 Glycaemic Status and Sarcopenia

As shown in Table 2, the prevalence of sarcopenia was higher among participants with diagnosed diabetes (27%) than among those without (13%), although this difference was not statistically significant (*p* = 0.08). However, the percentage with HbA1c ≥ 7.0% was significantly greater in the sarcopenia group (14%) than in the non‐sarcopenic group (8%; *p* = 0.009). Mean fasting glucose and HbA1c did not differ significantly between groups.

**TABLE 2:** Comparison of sarcopenia prevalence according to diabetes‐related indicators.

Variable	No sarcopenia (*n* = 1195)	Sarcopenia (*n* = 199)	*t*/χ^2^	*p*
Diabetes diagnosis, (%)	257 (22)	54 (27)	3.12	0.08
HbA1c ≥ 7%, *n* (%)	99 (8)	28 (14)	6.90	0.009
HbA1c (%), mean ± SD	5.92 ± 0.83	5.98 ± 0.79	−1.05	0.29
Fasting glucose (mg/dL)	107.07 ± 26.81	106.61 ± 22.00	0.26	0.79

Abbreviation: HbA1c, glycated haemoglobin.

### 3.3 Blood Pressure and Sarcopenia

Table 3 compares hypertension and blood pressure levels by sarcopenia status. The prevalence of physician‐diagnosed hypertension was nearly identical between groups (54.3% vs. 54.1%; *p* = 0.97). Although systolic blood pressure (SBP) did not differ significantly, diastolic blood pressure (DBP) was lower in the sarcopenia group (70.64 ± 8.81 mmHg) than in the non‐sarcopenic group (73.87 ± 8.70 mmHg; *p* < 0.001).

**TABLE 3:** Comparison of sarcopenia prevalence according to hypertension‐related indicators.

Variable	No sarcopenia (*n* = 1195)	Sarcopenia (*n* = 199)	*t*/χ^2^	*p*
Hypertension, *n* (%), yes	647 (54)	108 (54)	0.01	0.97
SBP (mmHg), mean ± SD	127.30 ± 16.28	126.06 ± 16.80	0.97	0.33
DBP (mmHg), mean ± SD	73.87 ± 8.70	70.64 ± 8.81	4.80	< 0.001

Abbreviations: BP, blood pressure; DBP, diastolic blood pressure; SBP, systolic blood pressure.

### 3.4 Factors Associated With Sarcopenia

Table 4 displays the results of the multivariable logistic regression analysis identifying factors independently associated with sarcopenia. After adjusting for covariates, age 76 years or older (OR = 3.19, 95% CI [2.27, 4.49], *p* < 0.001), alcohol consumption in the past year (O*R* = 0.58, 95% CI [0.41, 0.82], *p* = 0.002), meeting the WHO physical activity guideline (OR = 0.50, 95% CI [0.28, 0.90], *p* = 0.02) and BMI ≥ 25 kg/m^2^ (OR = 0.32, 95% CI [0.21, 0.48], *p* < 0.001) were significantly associated with sarcopenia. In addition, participants with HbA1c ≥ 7.0% were significantly more likely to have sarcopenia compared to those with lower levels (OR = 1.89, 95% CI [1.16, 3.08], *p* = 0.01). Other variables including sex, education, income, smoking and hypertension were not statistically significant predictors after adjustment.

**TABLE 4:** Factors associated with sarcopenia: Logistic regression analysis.

Variable	B	*p*	Exp (B)	95% CI for Exp (B)
Age ≥ 76 years	1.161	< 0.001	3.193	2.271–4.491
Sex (female)	0.134	0.46	1.144	0.804–1.627
Education ≤ elementary	0.631	0.12	2.004	0.867–4.119
Lowest income quartile	0.624	0.11	1.978	0.871–4.004
Current smoker (yes)	−0.102	0.75	0.903	0.483–1.691
Alcohol consumption (yes)	−0.547	0.002	0.579	0.408–0.820
WHO PA guideline (yes)	−0.699	0.02	0.497	0.276–0.895
Hypertension (yes)	−0.058	0.73	0.943	0.679–1.311
BMI ≥ 25 kg/m^2^	−1.14	< 0.001	0.320	0.214–0.479
HbA1c ≥ 7.0%	0.636	0.01	1.889	1.159–3.077

*Note:* The logistic regression model demonstrated a good fit to the data. The Nagelkerke R^2^ was 0.271, indicating that approximately 27% of the variance in sarcopenia status was explained by the model. The Hosmer–Lemeshow goodness‐of‐fit test was not statistically significant, χ^2^(8) = 5.21, *p* = 0.74, suggesting an adequate model fit. The model correctly classified 86% of participants.

Abbreviations: BMI, body mass index; CI, confidence interval; OR, odds ratio; PA, physical activity *p* < 0.05.

The logistic regression model demonstrated good fit to the data. The Nagelkerke *R* ^2^ was 0.271, indicating that approximately 27% of the variance in sarcopenia status was explained by the model. The Hosmer–Lemeshow goodness‐of‐fit test was not statistically significant, χ^2^(8) = 5.21, *p* = 0.74, suggesting adequate model fit. The overall classification accuracy was 86%.

## 4 Discussion

This study aimed to estimate the prevalence of sarcopenia among Korean older adults and examine its associations with glycaemic control, blood pressure and lifestyle factors using data from the 2023 Korea National Health and Nutrition Examination Survey (KNHANES). The prevalence of sarcopenia in our study population was 14%. Older age (76 years or older), poor glycaemic control (HbA1c ≥ 7.0%), lower BMI, physical inactivity and abstinence from alcohol consumption were significantly associated with sarcopenia. However, hypertension diagnosis and smoking were not significantly related to sarcopenia status.

### 4.1 Prevalence of Sarcopenia and Demographic Factors

The observed prevalence of sarcopenia (14%) aligns with previous reports from Asian community‐dwelling older adults, which generally range from 10% to 20% [14, 17]. It is also comparable to recent Korean studies, including analyses based on nationally representative data, which have reported prevalence estimates of approximately 13%–15% among older adults [15]. However, prevalence estimates vary across studies according to the diagnostic criteria and measurement methods applied. In particular, studies using different sarcopenia definitions and assessment tools have reported a wider range of prevalence. Such differences may be explained by variations in diagnostic criteria (e.g., AWGS 2014 vs. AWGS 2019), measurement techniques (bioelectrical impedance analysis vs. dual‐energy X‐ray absorptiometry) and study population characteristics, including age distribution and comorbidity burden [14, 15, 17]. Consistent with prior research, age was a strong predictor of sarcopenia [16]. Using ROC curve analysis, our study identified 76 years as the optimal age threshold for predicting sarcopenia, highlighting the increased vulnerability among individuals in their late 70s and beyond. Additionally, lower socioeconomic status, indicated by education and income, was significantly associated with sarcopenia, reflecting potential disparities in nutrition, health care access and lifestyle practices [18]. Thus, our findings reinforce the importance of age‐targeted screening and socioeconomically tailored interventions for sarcopenia prevention.

### 4.2 Glycaemic Control and Sarcopenia

Our results demonstrated that poor glycaemic control (HbA1c ≥ 7.0%) significantly increased the risk of sarcopenia, emphasising the negative impact of chronic hyperglycaemia on muscle health. This finding is consistent with evidence supporting a bidirectional relationship between sarcopenia and diabetes, where insulin resistance and sustained hyperglycaemia accelerate muscle protein degradation, subsequently worsening glucose metabolism [18, 19]. Although diabetes diagnosis has been reported as a risk factor for sarcopenia in some studies [7, 20], our findings did not show a statistically significant association. This discrepancy may reflect differences in population characteristics or in how diabetes was defined, as diagnosis can be based on self‐reported history, physician confirmation or medication use, whereas HbA1c provides an objective measure of current glycaemic control. Notably, poor glycaemic control (HbA1c ≥ 7.0%) remained a significant predictor, underscoring that glycaemic management, rather than diabetes status alone, may be more critical in preserving muscle mass and strength.

This interpretation is supported by recent studies showing that poor glycaemic control is associated with sarcopenia and low muscle mass in older adults with diabetes [19]. The level of HbA1c may therefore capture the cumulative effects of chronic metabolic dysregulation more directly than diabetes status alone. Chronic hyperglycaemia may promote muscle loss through multiple interrelated pathways, including insulin resistance, chronic inflammation, oxidative stress, impaired protein synthesis and reduced regenerative capacity of skeletal muscle [21]. Taken together, these findings suggest that glycaemic optimisation may have clinical importance not only for metabolic outcomes but also for maintaining muscle health, physical function and independence in later life.

### 4.3 Blood Pressure and Sarcopenia

In line with previous epidemiological studies, we found no significant association between hypertension diagnosis and sarcopenia [9]. However, an intriguing finding was that individuals with sarcopenia had lower diastolic blood pressure compared with counterparts without sarcopenia, despite similar systolic values. This may indicate reduced vascular compliance or underlying frailty, which are commonly observed in ageing populations with muscle loss, and warrants validation in longitudinal studies. Although some studies suggest that sarcopenia or sarcopenic obesity is linked with lower mean arterial pressure or reduced average blood pressure in non‐hypertensive older adults [22], other studies report that both SBP and DBP tend to increase with sarcopenia severity [23]. This inconsistency may reflect heterogeneity in study populations (hypertensive vs. non‐hypertensive; obesity status; ambulatory vs. clinic measurements) or underlying vascular changes, such as arterial stiffness or reduced compliance, that accompany ageing and muscle loss. Given the paucity and inconsistency of existing research on this topic, future longitudinal studies should investigate detailed haemodynamic patterns and vascular function to clarify the relationship between blood pressure and sarcopenia.

### 4.4 Behavioural and Lifestyle Factors

Our analysis confirmed the protective role of physical activity against sarcopenia, aligning with evidence that regular moderate‐intensity exercise preserves muscle mass and function in older populations [24, 25]. Interestingly, using alcohol consumption within the past year, participants who reported abstinence showed a higher prevalence of sarcopenia. This is consistent with a recent longitudinal study by [26], which reported a higher incidence of sarcopenia among non‐drinkers over 4 years [26]. However, many studies assessing long‐term drinking patterns have reported mixed or null associations. One possible explanation is reverse causality, as older adults with frailty or chronic conditions may refrain from alcohol. Alternatively, moderate alcohol intake may act as a proxy for healthier social interactions or lifestyle practices rather than providing a direct physiological benefit. Given potential confounding, cautious interpretation is necessary. Smoking status, meanwhile, showed no significant association with sarcopenia in our analysis, which aligns with prior evidence reporting inconsistent results: Although a meta‐analysis in Chinese older adults identified smoking as a significant risk factor [27], a US‐based study found no significant relationship between smoking status and muscle strength after adjustment for confounders [28]. These discrepancies may reflect differences in study design, population characteristics and the way smoking exposure is measured (e.g., current smoking vs. cumulative pack‐years).

### 4.5 Clinical Implications

Clinically, our findings highlight the importance of integrated geriatric care strategies targeting modifiable risk factors, particularly glycaemic management and physical activity. Routine screening for sarcopenia—especially in older adults with elevated HbA1c or limited physical activity—should be prioritised. Health providers could adopt multidisciplinary approaches, incorporating structured exercise programs, nutrition counselling and protein supplementation where appropriate, alongside comprehensive glycaemic control to mitigate sarcopenia risks and enhance quality of life in older adults.

### 4.6 Strengths and Limitations

Strengths include the use of nationally representative data, application of standardised AWGS 2019 criteria for sarcopenia, and ROC curve analysis to identify an age threshold predictive of sarcopenia. Limitations include the cross‐sectional design, which restricts causal inference; reliance on self‐reported variables (behavioural and chronic disease data), which may be subject to reporting bias; and potential residual confounding from unmeasured factors (e.g., inflammatory markers and hormone levels). In addition, information on the duration and timing of diagnosis of diabetes and hypertension was not available in the KNHANES dataset, which may have limited a more detailed understanding of the relationship between chronic disease progression and sarcopenia. Furthermore, bioelectrical impedance analysis was used instead of dual‐energy X‐ray absorptiometry, which may affect measurement accuracy.

## 5 Conclusions

This study highlights a substantial prevalence of sarcopenia among Korean older adults and identifies key associated factors, including older age, poor glycaemic control, physical inactivity, lower BMI and socioeconomic vulnerability. These findings emphasise the importance of tailored preventive interventions addressing glycaemic management, physical activity and nutritional strategies. Integrated, multidimensional approaches may help reduce sarcopenia risk and support healthy ageing in Korean society.

## Funding

The author has nothing to report.

## Ethics Statement

The Korea National Health and Nutrition Examination Survey (KNHANES) protocol was reviewed and approved by the Institutional Review Board of the Korea Disease Control and Prevention Agency (IRB No.: 2022‐11‐16‐R‐A). All participants provided written informed consent. This study analysed de‐identified, publicly available 2023 KNHANES data and required no additional ethics approval.

## Conflicts of Interest

The author declares no conflicts of interest.

## Data Availability

The datasets generated and analysed during the current study are publicly available from the Korea National Health and Nutrition Examination Survey (KNHANES) website: https://knhanes.kdca.go.kr.
